# A Bispecific Antibody That Simultaneously Recognizes the V2- and V3-Glycan Epitopes of the HIV-1 Envelope Glycoprotein Is Broader and More Potent than Its Parental Antibodies

**DOI:** 10.1128/mBio.03080-19

**Published:** 2020-01-14

**Authors:** Meredith E. Davis-Gardner, Barnett Alfant, Jesse A. Weber, Matthew R. Gardner, Michael Farzan

**Affiliations:** a Department of Microbiology and Immunology, The Scripps Research Institute, Jupiter, Florida, USA; Vaccine Research Center, NIH; Columbia University/HHMI

**Keywords:** broadly neutralizing antibodies, bispecific antibodies, human immunodeficiency virus 1, antibody neutralization, broadly neutralizing antibodies, human immunodeficiency virus

## Abstract

Broadly neutralizing antibodies (bNAbs) can prevent and control an HIV-1 infection, but their breadth is invariably too limited for use as monotherapy. To address this problem, bi- and trispecific antibody-like constructs have been developed. These engineered antibodies typically have greater breadth than the native bNAbs from which they were derived, but they are not more potent because they do not, in most cases, simultaneously engage more than a single epitope of the HIV-1 envelope glycoprotein (Env). Here, we describe a new class of bispecific antibodies targeting the V2-glycan (apex) and V3-glycan regions of the HIV-1 envelope glycoprotein (Env). Specifically, bispecific antibodies with a single-chain (scFv) form of the CAP256.VRC26.25 V2-glycan (apex) antibody on one antibody arm and a full V3-glycan Fab on the other arm neutralizes more HIV-1 isolates than the bNAbs from which they were derived. Moreover, these bispecific antibodies are markedly more potent than their parental bNAbs, likely because they simultaneously engage both the apex and V3-glycan epitopes of Env. Our data show that simultaneous engagement of two critical epitopes of a single Env trimer can markedly increase the potency of a bispecific antibody.

## Abstract (precis)

Broadly neutralizing antibodies (bNAbs) can prevent a new HIV-1 infection and can at least temporarily suppress an established infection. However, antibody-resistant viruses rapidly emerge in infected persons treated with any single bNAb. Several bispecific antibodies have been developed to increase the breadth of these antibodies, but typically only one arm of these bispecific constructs binds the HIV-1 envelope glycoprotein trimer (Env). Here, we develop and characterize bispecific constructs based on well-characterized V2-glycan and V3-glycan bNAbs and show that at least one member of this class is more potent than its parental antibodies, indicating that they can simultaneously bind both of these epitopes of a single Env trimer. These data show that bispecific antibody-like proteins can achieve greater neutralization potency than the bNAbs from which they were derived.

## INTRODUCTION

The past decade has seen the emergence of a number of potent broadly neutralizing antibodies (bNAbs) that recognize nearly every available surface of the HIV-1 envelope glycoprotein (Env) (1–7). Among these are antibodies that bind the CD4-binding site, the V1/V2 glycan region at the Env apex, the V3-glycan epitope, the interface of the gp120 and gp41 Env subunits, the silent face (SF), and in the membrane-proximal region of gp41 (8, 9). Although they are more potent and much broader than previously described HIV-1 neutralizing antibodies, the high HIV-1 mutation rate and preexisting diversity of Env in infected persons precludes their use as monotherapy (10–19). In many cases, their limited breadth also limits their use in prevention strategies. To address these limitations, bi- and multispecific antibody-like biologics of various architectures have been developed (20–23). Because they recognize two or more distinct epitopes, these inhibitors neutralize more HIV-1 isolates than the bNAbs from which they were derived. However, these constructs do not typically neutralize viruses more potently because they do not simultaneously engage more than one epitope on a single Env spike. They also do not in general cross-link two Env trimers because Env is sparsely distributed on the virion (15, 20–23).

Here, we characterize bispecific antibody-like constructs derived from two well-characterized classes of bNAbs. Specifically, we show that constructs composed a single-chain variable fragment-Fc (scFv-Fc) form of the V2-glycan antibody CAP256.VRC26.25 and one of several V3-glycan bNAbs neutralize a range of HIV-1 isolates more potently than either of its components. We further show that greater neutralization potency is likely a consequence of the ability of both arms of these bispecific antibodies to simultaneously engage a single Env trimer. Like other bispecific antibodies, these constructs also neutralize all isolates neutralized by either of the parental bNAbs. Our data suggest that greater breadth and potency can be obtained if bispecific antibodies are designed to simultaneously engage both of its Env epitopes.

## RESULTS

### Bispecific antibodies targeting the V2-apex and V3-glycan neutralize more efficiently than their parental antibodies.

**FIG 1:**
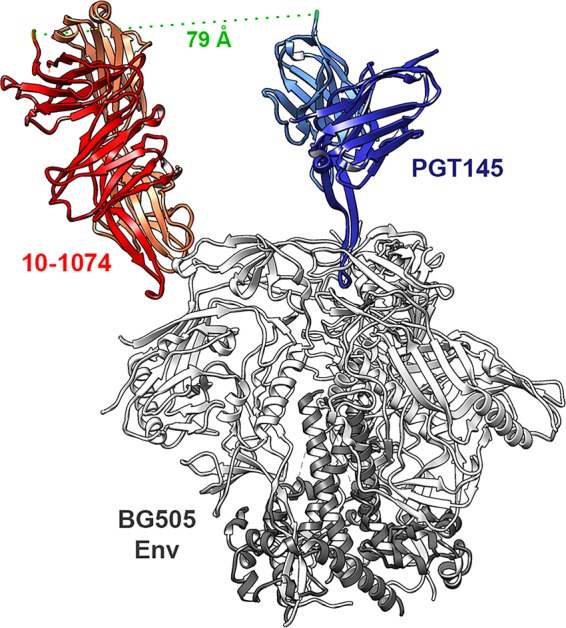
A model of V2-glycan and V3-glycan bNAbs bound to Env. The BG505 Env trimer bound to PGT145 (PDB accession no. 5V8L) was aligned with the BG505 SOSIP trimer complex with 10-1074 (PDB accession no. 5T3Z) Fab. BG505 Env is shown in white (gp120) and gray (gp41), PGT145 is shown in light (light chain) and dark (heavy chain) blue. 10-1074 is shown in light (light chain) and dark (heavy chain) red. The green dotted line connects the C terminus of the PGT145 light chain to the C terminus of the 10-1074 heavy chain, a 79-Å distance that is bridged by the linker and hinge regions of the Fc domain in the BISC variants described below.

**FIG 2:**
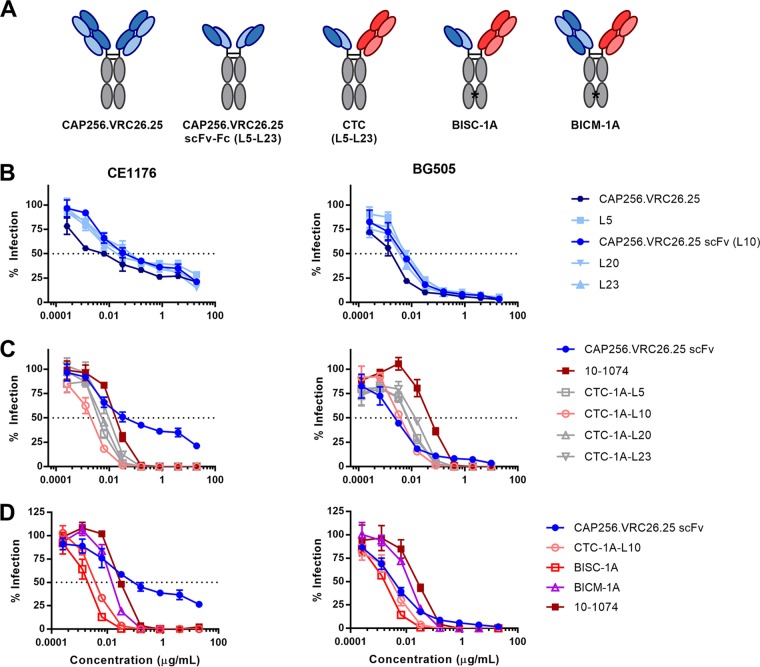
Bispecific constructs combining CAP256.VRC26.25 and 10-1074 are more potent than their parental antibodies. (A) Schematic representation of constructs used in this study. From left to right, wild-type antibody, a homodimeric scFv-Fc construct (e.g., CAP256.VRC26.25 scFv-Fc), a bispecific construct containing one scFv arm and one full antibody arm (i.e., CTM-1A-L10), a bispecific construct containing one scFv arm and one antibody arm with knob-in-hole (KIH) mutations (e.g., BISC-1A), a CrossMab construct of two full antibody arms with KIH mutations (i.e., BICR-1A). (B) TZM-bl neutralization curves of serial dilutions of wild-type CAP256.VRC26.25 (dark blue) or scFv-Fc constructs with various linker lengths against pseudotyped clade C CE1176 (left) and clade A BG505 (right) viruses. Infection is represented as the percentage of luciferase activity in the absence of inhibitor. Concentrations are shown as micrograms per milliliter. (C) TZM-bl neutralization curves as in panel B of cotransfected combinations (CTC) combining 10-1074 and CAP256.VRC26.25 scFv-Fc with various linker lengths. (D) TZM-bl neutralization curves as in panel B of bispecific constructs containing KIH mutations to stabilize heterodimerization. Values represent means ± standard errors of the means (SEM) (error bars) (*n* = 3). Data are representative of at least two independent experiments.

We speculated that by targeting two epitopes simultaneously, HIV-1 isolates would be neutralized more efficiently than antibodies targeting a single epitope. Inspection of cryoelectron microscopy (cryoEM) structures and models of the HIV-1 Env trimer bound to various neutralizing antibodies suggested that the Fc domains of V2-glycan class bNAbs and those of the V3 glycan class could be positioned to allow a bispecific antibody to bind both epitopes (Fig. 1). Accordingly, we investigated combinations of the V2-glycan bNAb CAP256.VRC26.25 with the V3-glycan bNAb 10-1074 (24, 25). Further modeling suggested that an scFv-Fc form of CAP256.VRC26.25 might better position the Fc domain of a bispecific antibody near the V3-glycan epitope than a complete Fab domain. We thus generated scFv-Fc and full-length antibody forms of CAP256.VRC26.25 and compared them in combination with full-length 10-1074 antibody, as represented in Fig. 2A. The linker separating the scFv and Fc domains was also varied, with different lengths of tetraglycine-serine linker (G_4_S) repeats. Homodimeric forms of these CAP256.VRC26.25 scFv-Fc constructs (L5, L10, L20, and L23, with the number indicating linker length and full nomenclature shown in Table 1) neutralized each of two HIV-1 isolates to comparable levels, albeit modestly less efficiently than the original CAP256.VRC26.25 bNAb (Fig. 2B). Combinations of each of these scFv-Fc constructs were then generated through cotransfection with plasmids expressing 10-1074 heavy and light chain, generating cotransfection combinations (CTC) of hetero- and homodimeric forms. These combinations neutralized the clade C isolate CE1176 more efficiently than either 10-1074 or an scFv-Fc form of CAP256.VRC26.25 with a 10-amino-acid linker (Fig. 2C). They also neutralized the clade A isolate BG505 isolate more efficiently than the 10-1074 antibody did and similarly to the CAP256.VRC26.25scFv-Fc homodimer. In contrast to these combinations, this scFv-Fc homodimer did not fully neutralize BG505. We also observed that mixtures, including a 10-amino acid scFv-Fc linker neutralized more efficiently than analogous mixtures with longer or shorter linkers. Because cotransfection combinations yielded both hetero- and homo-dimeric forms, we introduced knob-in-hole (KIH) mutations into the Fc domains to generate two additional constructs (26). BISC-1A (bispecific scFv) is a bispecific construct comprised of the CAP256.VRC26.25 single chain and 10-1074, whereas BICM-1A (bispecific cross Mab) is a bispecific construct of full-length versions of both antibodies using a cross-monoclonal antibody (Cross-Mab) architecture, ensuring that each light chain is associated with its own heavy chain (27). BISC-1A neutralized both CE1176 and BG505 more efficiently than the analogous cotransfection mixture (CTC-1A-L10), which in turn neutralized more efficiently than BICM-1A, the Cross-Mab form of 10-1074 and CAP256.VRC26.25 (Fig. 2D). All three combinations neutralized both isolates more efficiently than homodimeric forms of the CAP256.VRC26.25 scFv-Fc construct or the bNAb 10-1074. Notably, homodimeric CAP256.VRC26.25 scFv-Fc failed to completely neutralize CE1176 and neutralized BG505 less efficiently at higher concentrations, likely because it poorly neutralized a subset of Env conformational or glycosylation variants (28). In contrast, all combinations of CAP256.VRC26.25 and 10-1074 including CTC-1A-L10, BISC-1A, and BICM-1A neutralized these viruses completely. Thus, combinations of CAP256.VRC26.25 and 10-1074, and especially BISC-1A, neutralize these HIV-1 isolates markedly more efficiently than homodimeric 10-1074 or CAP256.VRC26.25 scFv-Fc.

**TABLE 1:** Nomenclature of bispecific constructs used in this study^*a*^

Construct^*b*^	Arm 1	Arm 2^*c*^	Linker^*d*^	Fc^*e*^
L5	CAP256.VRC26.25 scFv-Fc	n/a	5GS	WT
CAP256.VRC26.25 scFv-Fc	CAP256.VRC26.25 scFv-Fc	n/a	10GS	WT
L20	CAP256.VRC26.25 scFv-Fc	n/a	20GS	WT
L23	CAP256.VRC26.25 scFv-Fc	n/a	23GAE	WT
CTC-1A-L5	CAP256.VRC26.25 scFv-Fc	10-1074	4GS	WT
CTC-1A-L10	CAP256.VRC26.25 scFv-Fc	10-1074	10GS	WT
CTC-1A-L20	CAP256.VRC26.25 scFv-Fc	10-1074	20GS	WT
CTC-1A-L23	CAP256.VRC26.25 scFv-Fc	10-1074	23GAE	WT
BICM-1A	CAP256.VRC26.25 CrMab	10-1074	n/a	KIH
BISC-1A	CAP256.VRC26.25 scFv-Fc	10-1074	10GS	KIH
BISC-1B	CAP256.VRC26.25 scFv-Fc	PGT121	10GS	KIH
BISC-1C	CAP256.VRC26.25 scFv-Fc	PGT128	10GS	KIH
PGT145 scFv-Fc	PGT145 scFv-Fc	n/a	10GS	WT
BISC-2A	PGT145 scFv-Fc	10-1074	10GS	KIH
BISC-2B	PGT145 scFv-Fc	PGT121	10GS	KIH
BISC-2C	PGT145 scFv-Fc	PGT128	10GS	KIH

a The names and composition of the mono- and bispecific constructs characterized in this study are shown in the table.

b CTC indicates that a cotransfection combination of homo- and heterodimeric constructs was characterized. BICM indicates bispecific CrossMab, including full-length Fab forms of each bNAb. BISC indicates bispecific scFv-Fc construct comprised of an scFv-Fc and a full-length bNAb (Fab-Fc).

c n/a indicates that the construct Arm 2 is identical to its Arm 1.

d The linkers 5GS, 10GS, 20GS, and 23GAE are defined in Results and Materials and Methods, with the number indicating their length.

e WT indicates wild-type human IgG1 Fc domain, and KIH indicates knob-in-hole mutations that stabilize heterodimerization.

### CAP256-VRC26.25 scFv-Fc synergizes with multiple V3 glycan-targeting antibodies.

**FIG 3:**
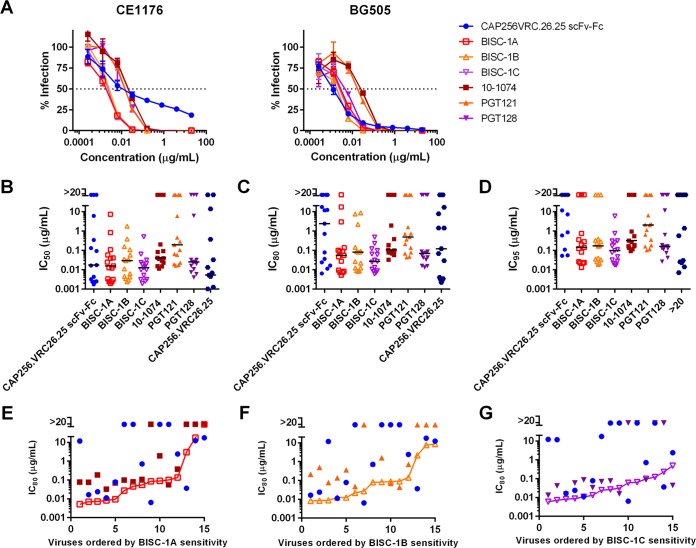
Bispecific constructs combining CAP256.VRC26.25 with V3 glycan antibodies are generally more potent than their parental antibodies. (A) TZM-bl neutralization curves as in Fig. 1. Curves are shown for viruses pseudotyped with CE1176 and BG505 for combinations of CAP256.VRC26.25 with 10-1074, PGT121, and PGT128. Values represent means ± SEM (*n* = 3). Data are representative of at least two independent experiments. (B to D) IC_50_ (B), IC_80_ (C), and IC_95_ (D) values are plotted for each isolate tested. Median neutralization efficiency values are indicated by black bars. Values represent means from two independent experiments of triplicates. (E to G) Comparison of bispecific construct potency to parental antibody potency. Viruses were ranked by sensitivity to the bispecofic constructs and IC_80_ values for parental antibodies and each BISC-1A (E), BISC-1B (F), and BISC-1C (G) were plotted against this ranking. Data points above the bispecific line indicate lower potency of the parental components compared to the bispecific construct.

We also investigated whether the enhanced neutralization observed with BISC-1A (the bispecific construct with CAP256.VRC26.25 scFv-Fc and 10-1074 arms) relative to its parental constructs could be extended to additional V3 glycan-targeting antibodies. We generated bispecific molecules consisting of the same CAP256.VRC26.25 scFv-Fc combined with full-length V3-glycan bNAbs PGT121 (BISC-1B) and PGT128 (BISC-1C) (7). These constructs were compared with each of their parental components for their ability to neutralize a 12-isolate global panel isolate and an additional 3 isolates (PVO4, ZM651, and BG505). Neutralization curves are shown for two isolates, CE1176 and BG505 that are differentially resistant to CAP256.VRC26.25 (Fig. 3A). Again, all three bispecific constructs neutralized these isolates more efficiently than any parental antibody, even when largely resistant to CAP256.VRC26.25. Fifty percent, 80%, and 95% inhibitory concentration (IC_50_, IC_80_, and IC_95_) values are plotted for all 15 isolates in Fig. 3B to D (numerical values are provided in Tables S1 to S3 in the supplemental material). Notably, unlike any of the parental antibodies, all bispecific constructs neutralized all 15 isolates with IC_50_ values less than 10 μg/ml (Fig. 3B and Table 2). Similarly, BISC-1B and BISC-1C neutralized all isolates with IC_80_ values less than 10 μg/ml, whereas the IC_80_ of BISC-1A for one isolate (CNE8) was greater than this concentration (Fig. 3C). Remarkably, BISC-1C neutralized all 15 isolates with IC_95_ values less than 10 μg/ml (Fig. 3D). Also, with two or three exceptions in each case, all bispecific constructs neutralized each isolate more efficiently than their parental antibodies (Fig. 3E to G). Bispecific constructs neutralized isolates that were sensitive (IC_80_ < 20 μg/ml) to both parental antibodies more efficiently than these parental antibodies (Table 3). Similarly, they neutralized isolates resistant to CAP256.VRC26.25 scFv-Fc and sensitive to the parental V3-glycan antibody more efficiently than their parental antibodies. However, CAP256.VRC26.25 scFv-Fc in general neutralized isolates that were resistant to V3-glycan bNAbs but sensitive to CAP256.VRC26.25, although these isolates were nonetheless efficiently neutralized in all cases. Notably, BISC-1C neutralized every isolate tested that was resistant (IC_80_ > 20 μg/ml) to both PGT128 and CAP256.VRC26.25. Thus, bispecific constructs combining CAP256.VRC26.25 and any of three V3-glycan antibodies neutralize the majority of a global panel of HIV-1 isolates more efficiently than any of their component antibodies.

**TABLE 2:** Viral coverage of CAP256.VRC26.25 bispecific constructs compared with their parental components^*a*^

Parameter (unit)	Value for parameter for construct:						
	CAP256-scFv^*b*^	10-1074	BISC-1A	PGT121	BISC-1B	PGT128	BISC-1C
Virus coverage for IC_50_ (no. of constructs [of 15 constructs])							
<20 μg/ml	12	11	15	11	15	12	15
<10 μg/ml	12	11	15	11	15	12	15
<1 μg/ml	11	11	14	10	14	11	15
<0.1 μg/ml	9	10	12	6	11	11	14
<0.01 μg/ml	6	0	5	0	6	3	6

Potency for IC_50_							
Median (μg/ml)	0.0172	0.0407	0.0157	0.1913	0.0293	0.0252	0.0128
Median (nM)	0.172	0.271	0.126	1.28	0.234	0.168	0.102
Geo mean (μg/ml)	0.0192	0.0363	0.0235	0.0248	0.0246	0.0248	0.0127
Geo mean (nM)	0.192	0.242	0.188	0.165	0.197	0.165	0.102
							
*P* value for IC_50_							
Compared to CAP256-scFv			0.359 (ns)		0.303 (ns)		0.041*
Compared to relevant V3-glycan bNAb			0.0012**		0.0084**		0.015*

Virus coverage for IC_80_ (no. of constructs [of 15 constructs])							
<20 μg/ml	11	11	14	11	15	11	15
<10 μg/ml	8	11	13	11	15	11	15
<1 μg/ml	7	10	12	10	12	11	15
<0.1 μg/ml	6	7	10	4	11	11	12
<0.01 μg/ml	1	0	5	0	3	0	4

Potency for IC_80_							
Median (μg/ml)	2.387	0.106	0.054	0.484	0.081	0.069	0.027
Median (nM)	23.87	0.707	0.432	3.23	0.648	0.460	0.216
Geo mean (μg/ml)	0.258	0.121	0.060	0.215	0.083	0.040	0.032
Geo mean (nM)	2.58	0.807	0.480	1.43	0.664	0.267	0.256

*P* value for IC_80_							
Compared to CAP256-scFv			0.121 (ns)		0.0043**		0.0026**
Compared to relevant V3-glycan bNAb			0.0006***		0.0004***		0.015*

a The number of isolates (of 15 isolates tested) sensitive to each construct at the indicated concentrations, median, and geometric (Geo) mean IC_50_ and IC_80_ values are provided in micrograms per milliliter and nanomolar. Statistical comparison of BISC constructs to parental component was determined by a Wilcoxon signed-rank test. Statistical significance; ns, not significant; *, *P* < 0.05; **, *P* < 0.005; ***, *P* < 0.001.

b CAP256-scFv, CAP256.VRC26.25 scFv-Fc.

**TABLE 3:** Viral sensitivity of CAP256.VRC26.25 bispecific constructs compared to parental components^*a*^

Sensitivity	*N*	IC_80_ (GM) (μg/ml)		
		**CAP256-scFv**^*b*^	**10-1074**	**BISC-1A**
CAP256.VRC26.25 (S) x 10-1074 (S)	7	0.183	0.099	0.015
CAP256.VRC26.25 (R) x 10-1074 (S)	4	>20	0.170	0.058
CAP256.VRC26.25 (S) x 10-1074 (R)	4	0.471	>20	1.627
All viruses	15	0.258	0.121	0.060

		**CAP256-scFv**	**PGT121**	**BISC-1B**
CAP256.VRC26.25 (S) x PGT121 (S)	7	0.183	0.297	0.020
CAP256.VRC26.25 (R) x PGT121 (S)	4	>20	0.122	0.062
CAP256.VRC26.25 (S) x PGT121 (R)	4	0.471	>20	1.398
All viruses	15	0.258	0.215	0.083

		**CAP256-scFv**	**PGT128**	**BISC-1C**
CAP256.VRC26.25 (S) x PGT128 (S)	9	0.484	0.042	0.020
CAP256.VRC26.25 (R) x PGT128 (S)	2	>20	0.034	0.029
CAP256.VRC26.25 (S) x PGT128 (R)	2	0.015	>20	0.117
CAP256.VRC26.25 (R) x PGT128 (R)	2	>20	>20	0.088
All viruses	15	0.258	0.040	0.032

a The geometric mean (GM) IC_80_ values for constructs grouped by susceptibility (S) or resistance (R) to individual parental components are provided.

b CAP256-scFv, CAP256.VRC26.25 scFv-Fc.

10.1128/mBio.03080-19.1 TABLE S1 IC_50_ values (μg/ml) of Cap256.VRC26.25 bispecific constructs. Download Table S1, DOCX file, 0.01 MB. Copyright © 2020 Davis-Gardner et al. 2020 Davis-Gardner et al. This content is distributed under the terms of the Creative Commons Attribution 4.0 International license.

Multiple mathematical models have been described to predict the neutralization potency of combinations of antibodies based on their observed IC_50_ and IC_80_ values (19, 29). We compared the Bliss-Hill and additive models of combinations of the BISC-1A components and equal mixtures of the parental components (Table S4). For 8 of the 15 isolates tested (CE1176, Zm651, x2278, CH119, BJOX2000, PV04, Tro11, and CE0217), BISC-1A performed better than either model or the experimentally determined 50:50 mixture. For the remaining seven isolates, including four fully resistant to 10-1074, the potency of BISC-1A was roughly equivalent to the models and mixture. Thus, BISC-1A demonstrates synergistic neutralization of some isolates, but even when synergy does not occur, it remains as potent as a mixture of its parental components.

10.1128/mBio.03080-19.3 TABLE S3 IC_95_ values (μg/ml) of Cap256.VRC26.25 bispecific constructs. Download Table S3, DOCX file, 0.01 MB. Copyright © 2020 Davis-Gardner et al. 2020 Davis-Gardner et al. This content is distributed under the terms of the Creative Commons Attribution 4.0 International license.

10.1128/mBio.03080-19.4 TABLE S4 IC_80_ values (μg/ml) of BISC-1A compared to models. 50:50 mix refers to equal mixtures of 10 μg/ml of each parental component. Bliss-Hill and Additive models were calculated using CombiNAber. Download Table S4, DOCX file, 0.01 MB. Copyright © 2020 Davis-Gardner et al. 2020 Davis-Gardner et al. This content is distributed under the terms of the Creative Commons Attribution 4.0 International license.

### Bispecific molecules targeting the V3-glycan epitope with an additional V2-apex epitope target.

**FIG 4:**
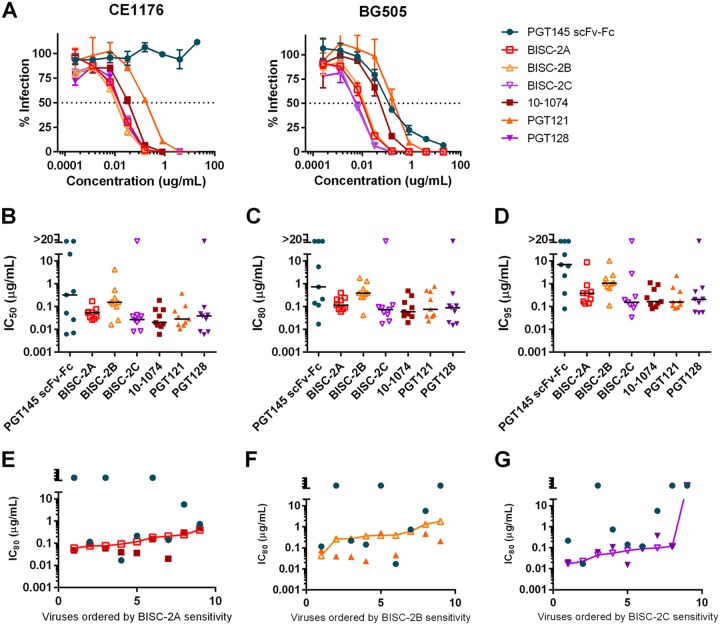
Characterization of bispecific constructs combining PGT145 with V3 glycan antibodies. (A) TZM-bl neutralization curves as in Fig. 1. Curves are shown for viruses pseudotyped with CE1176 and BG505 using combinations of PGT145 with 10-1074, PGT121, or PGT128. Values represent means ± SEM (*n* = 3). Data are representative of at least two independent experiments. (B to D) IC_50_ (B), IC_80_ (C), and IC_95_ (D) values are plotted for each isolate tested. Median neutralization efficiency values are indicated by black bars. Values represent means from two independent experiments of triplicates. (E to G) Comparison of bispecific construct potency to parental antibody potency. Viruses were ranked by potency to the parental antibodies and IC_80_ values for each parental component and BISC-2A (E), BISC-2B (F), and BISC-2C (G) were plotted against this ranking. Data points above the bispecific line indicate lower potency of the parental components compared to the bispecific construct.

To evaluate whether additional V2-glycan bNAbs could be combined with V3-glycan bNAbs, we performed a similar study using an scFv-Fc version of the V2-glycan bNAb PGT145 (7). Specifically, we generated constructs identical to BISC-1A, BISC-1B, and BISC-1C, except that the PGT145 scFv-Fc replaced that of CAP256.VRC26.25. Thus, BISC-2A, -2B, and -2C combined this scFv-Fc with 10-1074, PGT121, and PGT128, respectively. The CE1176 isolate is completely resistant to homodimeric PGT145 scFv-Fc, but combinations of the construct with 10-1074, PGT121, and PGT128 neutralized this isolate more efficiently than any V3-glyan bNAb (Fig. 4A). Similarly, BISC-2A and BISC-2B neutralized BG505 more efficiently than any of their parental antibodies, but BISC-2C neutralized with an efficiency similar to that of the potent neutralization of PGT128. However, these PGT145 bispecific constructs generally did not neutralize a nine-isolate subset of the panel characterized in Fig. 3 more efficiently than their parental antibodies, nor was their breadth greater than those of these antibodies (Fig. 4B to G, Tables 4 and 5, and Tables S5 to S7). We conclude that bispecific-based PGT145 scFv-Fc do not consistently neutralize more efficiently than their parental bNAbs and that distinct properties of CAP256.VRC26.25 contribute to the increased breadth and potency of BISC-1A, -1B, and -1C.

**TABLE 4:** Viral coverage of PGT145 bispecific constructs compared to parental components^*a*^

Parameter (unit)	Value for parameter for construct:						
	PGT145 scFv-Fc	10-1074	BISC-2A	PGT121	BISC-2B	PGT128	BISC-2C
Virus coverage for IC_50_ (no. of isolates [of 9 isolates])							
<20 μg/ml	6	9	9	9	9	8	8
<10 μg/ml	6	9	9	9	9	8	8
<1 μg/ml	4	9	9	9	8	8	8
<0.1 μg/ml	3	8	8	7	2	8	8
<0.01 μg/ml	0	1	0	0	0	3	2
							
Potency for IC_50_							
Median (μg/ml)	1.074	0.020	0.053	0.029	0.156	0.039	0.027
Median (nM)	10.74	0.133	0.423	0.191	1.25	0.258	0.213
Geo mean (μg/ml)	0.167	0.029	0.051	0.037	0.162	0.023	0.022
Geo mean (nM)	1.67	0.196	0.404	0.248	1.30	0.154	0.175

Virus coverage for IC_80_ (no. of isolates [of 9 isolates])							
<20 μg/ml	6	9	9	9	9	8	8
<10 μg/ml	5	9	9	9	9	8	8
<1 μg/ml	4	9	9	9	7	8	8
<0.1 μg/ml	1	6	4	5	1	5	7
<0.01 μg/ml	0	0	0	0	0	0	0

Potency for IC_80_							
Median (μg/ml)	2.78	0.059	0.116	0.076	0.392	0.087	0.073
Median (nM)	27.76	0.393	0.928	0.507	3.14	0.580	0.584
Geo mean (μg/ml)	0.861	0.082	0.134	0.117	0.395	0.058	0.054
Geo mean (nM)	8.61	0.547	1.07	0.780	3.16	0.387	0.432

a The number of isolates (of nine isolates tested) sensitive to each construct at the indicated concentrations, median, and geometric (Geo) mean IC_50_ and IC_80_ values are provided in micrograms per milliliter.

**TABLE 5:** Viral sensitivity of PGT145 bispecific constructs compared to parental components^*a*^

Sensitivity	*N*	IC_80_ (GM) (μg/ml)		
		**PGT145 scFv-Fc**	**10-1074**	**BISC-2A**
PGT145 scFv-Fc (S) x 10-1074 (S)	6	0.861	0.085	0.160
PGT145 scFv-Fc (R) x 10-1074 (S)	3	>20	0.075	0.094
All viruses	9	0.861	0.082	0.134

		**PGT145 scFv-Fc**	**PGT121**	**BISC-2B**
PGT145 scFv-Fc (S) x PGT121 (S)	6	0.861	0.101	0.329
PGT145 scFv-Fc (R) x PGT121 (S)	3	>20	0.159	0.570
All viruses	9	0.861	0.117	0.395

		**PGT145 scFv-Fc**	**PGT128**	**BISC-2C**
PGT145 scFv-Fc (S) x PGT128 (S)	6	0.861	0.051	0.049
PGT145 scFv-Fc (R) x PGT128 (S)	2	>20	0.082	0.072
PGT145 scFv-Fc (R) x PGT128 (R)	1	>20	>20	>20
All viruses	9	0.861	0.058	0.054

a Geometric mean (GM) IC_80_ values for constructs grouped by susceptibility (S) or resistance (R) to individual parental components.

10.1128/mBio.03080-19.5 TABLE S5 IC_50_ values (μg/ml) of PGT145 bispecific constructs. Download Table S5, DOCX file, 0.01 MB. Copyright © 2020 Davis-Gardner et al. 2020 Davis-Gardner et al. This content is distributed under the terms of the Creative Commons Attribution 4.0 International license.

### BISC-1A simultaneously engages V2- and V3-glycan epitopes of the Env trimer.

**FIG 5:**
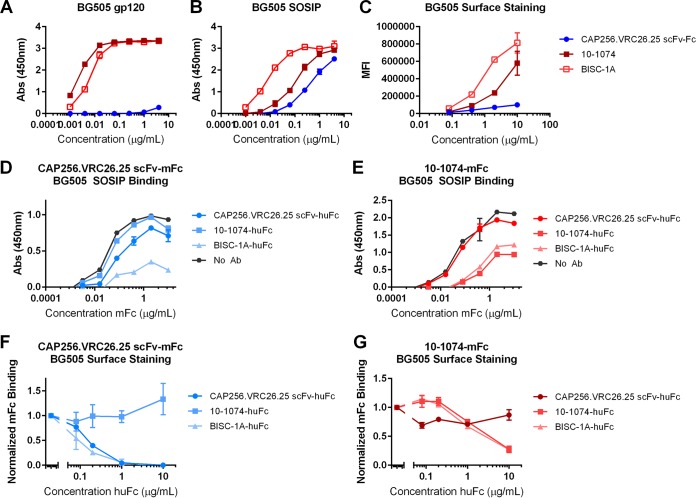
A bispecific construct BISC-1A binds BG505 SOSIP trimers and cell-expressed Env more efficiently than its components. (A and B) Env binding ELISAs. Plates were coated with BG505 gp120 (A) or BG505 SOSIP trimer (B) and then incubated with serial dilutions of the indicated antibodies or BISC-1A. Binding was detected with an HRP-conjugated secondary antibody. The absorbance at 450 nm is shown on the *y* axes in panels A and B. Data are representative of two independent experiments, and error bars represent range. (C) HEK293T cells were transfected to express BG505 Env lacking its cytoplasmic tail (BG505 ΔCT). Cells were harvested and stained with CAP256.VRC26.25 scFv, 10-1074, or BISC-1A. Binding was determined by flow cytometry with a FITC-conjugated secondary antibody. MFI, mean fluorescence intensity. (D and E) BG505 SOSIP trimer-coated ELISA plates were preincubated with 1 μg/ml of the constructs indicated in the legend. Binding of serial dilutions CAP256.VRC26.25-scFv-mFc (D) or 10-1074-mFc (E) was then measured. Baseline BG505 SOSIP binding, in the absence of a competing human antibody, is shown in gray (no antibody [No Ab]). Data are representative of two independent experiments, and error bars represent range. (F and G) HEK293T cells were transfected to express BG505 ΔCT. Cells were harvested and preincubated with serial dilutions of human Fc antibodies or BISC-1A before being stained with CAP256.VRC26.25-scFv-mFc (F) or 10-1074-mFc (G). Binding was determined by flow cytometry with an APC-conjugated secondary antibody. Data are representative of two independent experiments, and error bars represent range.

The increased potency of BISC-1A, -1B, and -1C relative to their respective parental bNAbs suggested that both arms of these bispecific constructs could simultaneously engage a single Env trimer. To determine whether this were the case, we compared binding of BISC-1A and its parental components for their ability to associate with monomeric gp120 of the BG505 isolate, soluble trimeric SOSIP Env of the same isolate, and cells transfected to expressed BG505 Env truncated in its cytoplasmic domain to facilitate efficient expression on the cell surface. 10-1074 bound monomeric gp120 more efficiently than BISC-1A, consistent with the dependence of the CAP256.VRC26.25 on a quaternary epitope (Fig. 5A). In contrast, BISC-1A bound BG505 SOSIP trimers markedly more efficiently than either of its parental constructs (Fig. 5B), indicating that both arms of the bispecific construct were simultaneously engaged. The marked difference between BISC-1A and 10-1074 could also be observed with cell-expressed BG505 Env (Fig. 5C). Competition binding studies, both enzyme-linked immunosorbent assay (ELISA) studies of SOSIP trimers and surface staining of cell-expressed Env, were also consistent with BISC-1A engaging both of its Env epitopes. For the ELISAs, plates were coated with BG505 SOSIP trimer and then incubated with a fixed 1-μg/ml concentration of CAP256.VRC26.25-scFv-Fc, 10-1074, or BISC-1A. The plates were washed and then incubated with serial dilutions of either CAP256.VRC26.25-scFv-Fc (Fig. 5D) or 10-1074 (Fig. 5E) with a mouse Fc domain. Binding of the mouse Fc proteins was determined. Compared to the baseline binding in the absence of a competing antibody (gray), CAP256.VRC26.25-scFv-mFc binding was inhibited by both CAP256.VRC26.25-scFv-huFc and BISC-1A. Similarly, 10-1074-mFc was inhibited by both 10-1074-huFc and BISC-1A. There was minimal cross-competition between the parental antibodies. Similar results were obtained with surface staining (Fig. 5F and G). In this case, cells expressing BG505 ΔCT (cytoplasmic tail) were preincubated with various concentrations of the human constructs and subsequently stained with a fixed amount of the mouse Fc (mFc) constructs. Binding as determined by flow cytometry was normalized to the baseline without preincubation. As observed with ELISA binding, the parental antibodies were inhibited by BISC-1A and their human Fc (hu-Fc) versions. Combined with enhanced neutralization potency of BISC-1A relative to the parental bNAbs, the data of Fig. 5 indicate that BISC-1A, and likely BISC-1B and BISC-1C, simultaneously bind both the V2-glycan and the V3-glycan epitopes of a single Env.

### BISC variants are broader and more potent than their parental components.

**FIG 6:**
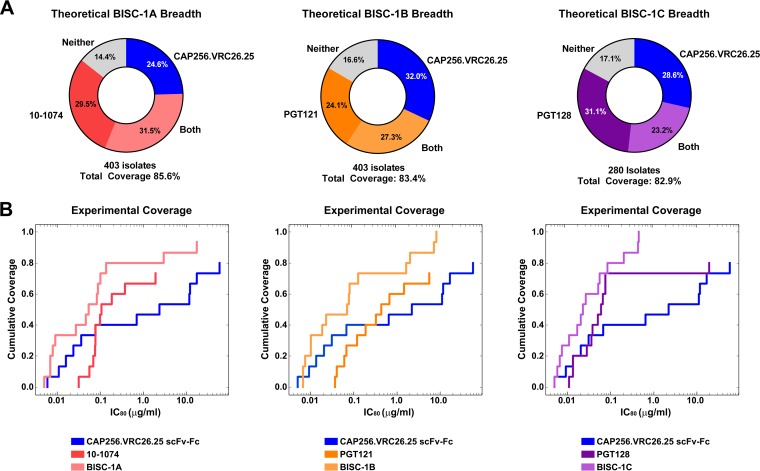
Theoretical breadth of BISC constructs from individual neutralization data available on CATNAP. (A) Using the CATNAP database, we analyzed the number of isolates neutralized by each component of BISC-1A (CAP256.VRC26.25 and 10-1074), BISC-1B (CAP256.VRC26.25 and PGT121), and BISC-1C (CAP256.VRC26.25 and PGT128) using an IC_80_ of <20 μg/ml as the cutoff. These results are represented as pie charts showing the percentage of isolates neutralized by each antibody individually, as well as those neutralized by both antibodies. Below each chart is the total number of isolates included in the analysis and the theoretical coverage percentage of viruses neutralized by at least one of the component antibodies. (B) Potency-breadth analysis of experimentally determined IC_80_ neutralization values provided in Table S2 in the supplemental material for the indicated constructs as analyzed with CombiNAber online tools.

We determined a theoretical breadth of BISC-1A, -1B, and -1C by analyzing all isolates for which neutralizing data were available for CAP256.VRC26.25 and each of the V3-glycan antibodies used in these bispecific constructs (15, 19, 20, 22–25, 29–35). As shown in Fig. 6A, 403 isolates have been studied with both CAP256.VRC26.25 and 10-1074. Of these isolates, 226 or 56.1% are neutralized by CAP256.VRC26.25 with IC_80_ values of less than 20 μg/ml. Similarly, 246 or 61.0% are neutralized by 10-1074, but 345 or 85.6% are neutralized by at least one of these bNAbs, suggesting that BISC-1A would neutralize approximately this proportion of isolates. Similarly, 336 of 403 or 83.4% would theoretically be neutralized by BISC-1B ,and 232 of 280 (82.9%) would be neutralized by BISC-1C. Thus, these constructs are likely to neutralize a significantly wider range of isolates than CAP256.VRC26.25, 10-1074, PGT121, or PGT128. We then generated a potency-breadth plot of the observed neutralization efficiency for BISC-1A, BISC-1B, and BISC-1C. When experimentally determined IC_80_ values from Table S2 were plotted for the 15 isolates tested in this study, each BISC construct had both increased breadth and increased potency compared to the parental components. Thus, BISC constructs neutralize a larger fraction of isolates than their components, and they do so more efficiently.

10.1128/mBio.03080-19.2 TABLE S2 IC_80_ values (μg/ml) of Cap256.VRC26.25 bispecific constructs. Download Table S2, DOCX file, 0.01 MB. Copyright © 2020 Davis-Gardner et al. 2020 Davis-Gardner et al. This content is distributed under the terms of the Creative Commons Attribution 4.0 International license.

## DISCUSSION

In this study, we examined the neutralization potency of bispecific molecules targeting the V2 apex and the V3 glycan sites on HIV-1 Env. We found that targeting a combination of these epitopes led to increased neutralization of a global panel of viruses. Specifically, heterodimeric combinations of CAP256.VRC26.25-scFv-Fc with any of three V3-glycan antibodies neutralized most viruses tested with markedly greater efficiency than homodimeric forms of their components. These observations contrast with most other reported bi- and multispecific bNAb constructs (20–23). For example, Asokan et al. reported greater breadth, but no increase in potency when a panel of bNAb combinations were characterized (20). Similarly, bispecific and trispecific constructs reported by Khan et al. (21) and Steinhardt et al. (22), respectively, were not more potent, but again much broader, reflecting the sum of isolates neutralized by their component antibodies. One exception is the study of Bournazos et al. who observed greater neutralization when bNAbs were combined and a 62-amino-acid IgG3-derived linker was used to connect the Fab regions to the Fc domains (23). That study also observed greatest synergy when 3BNC117 and a less well-characterized V3-glycan antibody, PGT135, were combined but did not observe enhanced neutralization when 3BNC117 was combined with either 10-1074 or PGT128, both of which are markedly broader than PGT135 (7). The greater enhancement, higher potency, and more compact structure of BISC-1A, -1B, and -1C is consistent with the greater proximity of the V2- and V3-glycan epitopes compared with the epitopes targeted in the study of Bournazos et al. In addition, we were uniquely able to demonstrate greater binding of our bispecific constructs to SOSIP trimers and to cell-expressed Env, consistent with their higher potency.

We also observed that combinations of 10-1074 and an scFv-Fc form of CAP256.VRC26.25 neutralized more efficiently than the same combination in CrossMab format (Fig. 2). CAP256.VRC26.25 has not been solved in complex with Env. However, a structure of PGT145 bound to the Env trimer, used in Fig. 1, indicates that the C terminus of its scFv would be positioned closer than the C1 domain of the full-length antibody to the C terminus of the 10-1074 C1 domain. Nonetheless, we did not observe synergy when a PGT145 scFv-Fc was combined with 10-1074 using an identical 10-amino-acid linker, implying that the CAP256.VRC26.25 scFv is more closely positioned to the C1 domains of V3-glycan antibodies. Perhaps a longer linker or a different arrangement of scFv components might also allow PGT145 scFv-Fc to synergize with V3-glycan antibodies.

One limitation of V2-glycan antibodies is their frequent inability to completely neutralize otherwise sensitive HIV-1 isolates, reflected in the fact that their IC_80_ and especially IC_95_ values are higher than their IC_50_ values would indicate (Fig. 3A and 4A) (28). Incomplete neutralization remains a concern with this class of antibodies because it could accelerate the emergence of resistance. Notably, we consistently observed complete neutralization with our BISC variants despite the fact that CAP256.VRC26.25 scFv significantly contributed to neutralization. Thus, the bispecific variants described here do not share this limitation of V2-glycan antibodies.

It has previously been reported that one V2-glycan antibody, PG9, can interfere with neutralization by V3-glycan antibodies in some circumstances (29). Similar interference may account for the inability of PGT145 to combine usefully with V3-glycan antibodies in the BISC2 constructs characterized in Fig. 4. It may also suggest that the Cross-Mab construct BICM-1A is less potent than BISC-1A because the Fab of the former construct interferes with binding of its 10-1074 arm. A structure of CAP256.VRC26.25 in complex with Env may clarify the basis of this interference and facilitate the development of more potent variants. It is also unclear whether the BISC variants have pharmacokinetic (PK) properties that are similar to those of their component molecules, and the half-lives of bispecific and multispecific antibodies can frequently be limited by their shorter-half-life arm. Further evaluation and optimization of these constructs *in vivo* will be necessary before they might be considered for clinical use. Such optimization may be warranted: BISC-1A, -1B, and -1C are broader and more potent than either CAP256.VRC26.25 or their respective 10-1074, PGT121, and PGT128 component bNAbs. The architecture of these constructs allows for straightforward production in cell culture. Perhaps most importantly, they demonstrate that significant synergy can be obtained when two proximal Env epitopes are simultaneously engaged.

## MATERIALS AND METHODS

### Cells and plasmids.

HEK293T (ATCC, Manassas, VA) and TZM-bl cell lines were grown in Dulbecco modified Eagle medium (DMEM) supplemented with 10% fetal bovine serum. TZM-bl cells were obtained through the NIH AIDS Reagent Program, Division of AIDS, NIAID, NIH, contributed by John C. Kappes, Xiayun Wu, and Tranzyme Inc. (36–40). Expi293F cells were grown in Expi293 expression medium (Life Technologies, Carlsbad, CA).

The variable heavy and light chains of PGT145 were cloned into human IgG1 expression vectors as previously described (41). 10-1074 expression plasmids were provided by Michel Nussensweig. PGT121 and PGT128 were provided by Dennis Burton. Heavy and light chains for PGT145 were synthesized by Integrated DNA Technologies (IDT) (Newark, NJ) and cloned into IgG1 expression vectors. scFv-Fc versions of Cap256-VRC26.25 and PGT145 were constructed by cloning the variable heavy and variable light chains separated by a 10-residue G_4_S linker fused to an IgG1 Fc domain. Different linkers were tested to separate the scFv and Fc domains, including 5-, 10-, and 20-residue G_4_S linkers and a 23-residue linker designed to limit glycosylation sites (amino acid sequence, GGAGGEAGAGGAGGAGGEAGAGG). Knobs-in-holes mutations were generated by mutation of S354C/T366W in the V3-glycan antibodies (“knobs”) and Y349C/T366S/L368A/Y407V in the apex antibodies and scFv-Fc (“holes”) (26). For competition staining, the 10-1074 variable heavy chain and CAP256.VRC26.25 scFv were cloned into murine IgG2a Fc expression vectors. Expression vectors for the BG505 gp160-Δcytoplasmic tail were provided by John Moore and P. J. Klasse.

### Protein production and purification.

Antibodies were produced in Expi293 cells (Life Technologies, Carlsbad, CA). Cells were grown to a density of 3 × 10^6^ cells/ml prior to transfection with FectoPRO according to the manufacturer’s instructions (Polyplus, New York, NY). Total DNA (140 μg) was transfected in 250 ml Expi293 cells. Antibodies were produced by transfection of two plasmids encoding heavy and light chain, respectively, at a 1:1 ratio. Constructs using CAP256-VRC26.25 and PGT145 plasmids were cotransfected at an 4:1 ratio with plasmid encoding human tyrosine protein sulfotransferase 2 (TPST2) to ensure proper sulfation of CDRH3 residues. Bispecific constructs were produced by transfection of a 4:2:3:1 ratio of scFv-Fc:Heavy:Light:TPST2 plasmids. Five days posttransfection, the medium was collected for protein purification. Debris was cleared by centrifugation for 10 min at 4,000 × *g* and filtered using 0.45-μm filter flasks (Thermo Scientific, Waltham, MA). Antibodies and Fc-fusion proteins were purified from supernatants using HiTrap MabSelect SuRe columns (GE Healthcare Life Sciences, Pittsburgh, PA). After protein binding, columns were washed extensively with phosphate-buffered saline (PBS) before elution with IgG elution buffer (Thermo Scientific, Waltham, MA). Eluate pH was immediately adjusted with Tris-HCl 1 M pH 9.0 neutralization buffer (G-Biosciences, St. Louis, MO). Buffer was exchanged with PBS, and protein was concentrated to 1 mg/ml by ultrafiltration (Amicon Ultra, Millipore Sigma, Billerica, MA) at 4,000 × *g*. Protein concentration was measured using the IgG setting (molar extinction coefficient of 210,000 M^−1^ cm^−1^) on a Nanodrop spectrophotometer (Thermo Scientific, Waltham, MA).

BG505 SOSIP trimer was produced by transfection of Expi293 cells with BG505.SOSIP.664 gp140 containing a His tag and pFurin plasmid. Supernatants were harvested after 96 h and initially purified on HIS-trap columns (GE Healthcare Life Sciences, Pittsburgh, PA). Trimer was further purified using PGT145 coupled to CNBr-activated Sepharose 4B (GE Healthcare Life Sciences, Pittsburgh, PA) according to the manufacturer’s instructions (42). Trimer was eluted with 3 M MgCl_2_, and immediately buffer was exchanged with PBS and protein was concentrated to 1 mg/ml by ultrafiltration (Amicon Ultra, Millipore Sigma, Billerica, MA) at 4,000 × *g*.

### Pseudotyped viruses.

HEK293T cells were transiently transfected with 45 μg of a plasmid expressing Env of the indicated HIV-1 isolate and 45 μg of pNL4.3ΔEnv, an HIV-1 expression vector lacking a functional *env* gene. Medium was changed at 12 h posttransfection. At 48 h after the medium change, supernatants were collected and filtered through a 0.45-μm syringe filter. Aliquoted pseudoviruses were stored at −80°C.

### TZM-bl neutralization assay.

TZM-bl neutralization assays were performed as previously described (41, 43, 44). Briefly, antibody titrations were incubated with pseudotyped viruses for 1 h at 37°C. TZM-bl cells were diluted in DMEM to 100,000 cells/ml and added to the virus/inhibitor mix. Cells were then incubated for 48 h at 37°C. Viral entry was determined by luciferase readout with BriteLite Plus (Perkin Elmer, Waltham, MA) and read on a Victor X3 plate reader (Perkin Elmer, Waltham, MA).

### ELISA.

Enzyme-linked immunosorbent assay (ELISA) plates (Costar) were coated with 5 μg/ml HIV-1 gp120 (Immune Technology Corp.) or BG505.664.SOSIP trimer and left overnight at 4°C. The plates were washed with PBS plus 0.05% Tween 20 (PBS-T) twice and blocked with 5% bovine serum albumin (BSA) in PBS for 1 h at 37°C. Dilutions of antibodies blocked with 5% BSA in PBS were added to the plate and incubated for 1 h at 37°C. Samples were washed five times with PBS-T and labeled with a horseradish peroxidase-conjugated secondary antibody (Jackson Immuno Research) recognizing human IgG1. Plates were incubated for 1 h at 37°C and then washed 10 times with PBS-T. Tetramethylbenzidine (TMB) solution (Fisher) was added and left for 10 min at room temperature, and then the reaction was stopped with TMB stop solution (Southern Biotech). Absorbance was measured at 450 nm with a Victor X3 plate reader (Perkin Elmer).

For competition binding, ELISA plates were coated with BG505.664.SOSIP trimers and left overnight at 4°C. The plates were washed with PBS plus 0.05% Tween 20 (PBS-T) twice and blocked with 5% BSA in PBS for 1 h at 37°C. A saturating concentration (1 μg/ml) of antibodies with human Fc domains, blocked with 5% BSA in PBS, was added to the wells of the plate and incubated for 1 h at 37°C. Samples were washed five times with PBS-T before the addition of serial dilutions of CAP256.VRC26.25 scFv-mouse-Fc or 10-1074 with a mouse Fc and incubated for 1 h at 37°C. Samples were washed five times with PBS-T and labeled with a horseradish peroxidase-conjugated secondary antibody (Jackson Immuno Research) recognizing mouse IgG. Plates were incubated for 1 h at 37°C and then washed 10 times with PBS-T. Tetramethylbenzidine (TMB) solution (Fisher) was added and left for 10 min at room temperature, and then the reaction was stopped with TMB stop solution (Southern Biotech). Absorbance was measured at 450 nm with a Victor X3 plate reader (PerkinElmer).

### HIV Env surface staining assay.

HEK293T cells were transfected with plasmids expressing the HIV-1 BG505 envelope glycoprotein lacking cytoplasmic residues 732 to 876 (HXBc2 numbering), BG505 ΔCT (cytoplasmic tail). Cells were collected 48 h posttransfection with nonenzymatic dissociation buffer (Sigma-Aldrich, St. Louis, MO). Cells were washed with flow cytometry buffer (PBS with 2% goat serum, 0.01% sodium azide) before incubation with antibodies for 1 h on ice. Cells were washed twice with flow cytometry buffer. Antibody binding was determined with allophycocyanin (APC)-conjugated goat anti-human secondary antibodies (Jackson ImmunoResearch, West Grove, PA). After incubation with secondary antibody, cells were washed once with flow cytometry buffer, once with PBS, and then resuspended in 1% paraformaldehyde in PBS. Binding was analyzed with an Accuri C6 flow cytometer, and data were analyzed with the C6 Software (BD Biosciences, San Jose, CA).

For competition binding, HEK293T cells were transfected with plasmids expressing BG505 ΔCT. Cells were collected 48 h posttransfection with nonenzymatic dissociation buffer (Sigma-Aldrich, St. Louis, MO). Cells were washed with flow cytometry buffer (PBS with 2% goat serum, 0.01% sodium azide) before incubation with serial dilutions of human Fc domain containing scFv-Fc, antibody, or BISC-1A for 1 h on ice. Cells were washed twice with flow cytometry buffer, and incubated with 200 ng/ml of mouse Fc containing CAP256.VRC26.25 scFv-mouse-Fc or 10-1074 with a mouse Fc. Antibody binding was determined with APC-conjugated goat anti-human and fluorescein isothiocyanate (FITC)-conjugated goat anti-mouse secondary antibodies (Jackson ImmunoResearch, West Grove, PA). After incubation with secondary antibody, cells were washed once with flow cytometry buffer, once with PBS, and then resuspended in 1% paraformaldehyde in PBS. Binding was analyzed with an Accuri C6 flow cytometer, and data were analyzed with C6 software (BD Biosciences, San Jose, CA).

### Modeling and predictions.

Predicted potency of combinations of antibodies and potency-breadth curves were calculated using the Los Alamos National Labs CombiNAber tool (19, 29).

### Statistical analysis.

Wilcoxon signed-rank test analysis was performed in GraphPad Prism.

10.1128/mBio.03080-19.6 TABLE S6 IC_80_ values (μg/ml) of PGT145 bispecific constructs. Download Table S6, DOCX file, 0.01 MB. Copyright © 2020 Davis-Gardner et al. 2020 Davis-Gardner et al. This content is distributed under the terms of the Creative Commons Attribution 4.0 International license.

10.1128/mBio.03080-19.7 TABLE S7 IC_95_ values (μg/ml) of PGT145 bispecific constructs. Download Table S7, DOCX file, 0.01 MB. Copyright © 2020 Davis-Gardner et al. 2020 Davis-Gardner et al. This content is distributed under the terms of the Creative Commons Attribution 4.0 International license.
