# Supplementary material for: A Bispecific Antibody That Simultaneously Recognizes the V2- and V3-Glycan Epitopes of the HIV-1 Envelope Glycoprotein Is Broader and More Potent than Its Parental Antibodies
Source: mBio. 2020 Jan 14;11(1):e03080-19. doi: 10.1128/mBio.03080-19 (PMC6960291; doi:10.1128/mBio.03080-19)
Supplement: TABLE S1 [file mBio.03080-19-st001.docx]

**Table S1. IC_50_ Values (µg/mL) of Cap256.VRC26.25 Bispecific Constructs.**

|  | **IC_50_s** | | | | | | | |
| --- | --- | --- | --- | --- | --- | --- | --- | --- |
|  | **CAP256.**  **VRC26.25** | **10-1074** | **PGT121** | **PGT128** | **CAP256.VRC26.25 scFv-Fc** | **BISC-1A** | **BISC-1B** | **BISC-1C** |
| **CE1176** | 0.004 | 0.023 | 0.023 | 0.016 | 0.017 | 0.002 | 0.003 | 0.003 |
| **Zm651** | 0.372 | 0.091 | 0.461 | >20 | 6.030 | 0.039 | 1.776 | 0.496 |
| **x2278** | 0.001 | 0.029 | 0.104 | 0.011 | 0.003 | 0.003 | 0.005 | 0.005 |
| **BG505** | 0.001 | 0.015 | 0.058 | 0.007 | 0.002 | 0.002 | 0.003 | 0.003 |
| **CH119** | 0.013 | 0.039 | 0.281 | 0.033 | 0.093 | 0.024 | 0.035 | 0.031 |
| **BJOX2000** | 0.001 | 0.011 | 0.065 | 0.020 | 0.003 | 0.003 | 0.004 | 0.020 |
| **25710** | 0.000 | 0.082 | 0.191 | 0.025 | 0.002 | 0.002 | 0.002 | 0.003 |
| **PV04** | 0.033 | 0.199 | 5.875 | 0.025 | 0.343 | 0.057 | 0.058 | 0.063 |
| **Tro11** | >20 | 0.036 | 0.016 | 0.032 | >20 | 0.012 | 0.029 | 0.014 |
| **CNE8** | 0.007 | >20 | >20 | 0.023 | 0.130 | 7.280 | 0.384 | 0.010 |
| **CNE55** | 0.001 | >20 | >20 | >20 | 0.004 | 0.109 | 0.109 | 0.019 |
| **x1632** | 0.001 | >20 | >20 | >20 | 0.002 | 0.013 | 0.006 | 0.010 |
| **246F3** | 0.005 | >20 | >20 | 0.004 | 0.016 | 0.821 | 0.155 | 0.002 |
| **398F1** | >20 | 0.015 | 0.019 | 0.006 | >20 | 0.040 | 0.033 | 0.013 |
| **CE0217** | 13.700 | 0.041 | 0.018 | 6.100 | >20 | 0.016 | 0.011 | 0.024 |
