# Supplementary material for: A Bispecific Antibody That Simultaneously Recognizes the V2- and V3-Glycan Epitopes of the HIV-1 Envelope Glycoprotein Is Broader and More Potent than Its Parental Antibodies
Source: mBio. 2020 Jan 14;11(1):e03080-19. doi: 10.1128/mBio.03080-19 (PMC6960291; doi:10.1128/mBio.03080-19)
Supplement: TABLE S3 [file mBio.03080-19-st003.docx]

**Table S3. IC_95_ Values (µg/mL) of Cap256.VRC26.25 Bispecific Constructs.**

|  | **IC_95_s** | | | | | | | | | |
| --- | --- | --- | --- | --- | --- | --- | --- | --- | --- | --- |
|  | **CAP256.**  **VRC26.25** | **10-1074** | | **PGT121** | **PGT128** | | **CAP256.**  **VRC26.25 scFv-Fc** | **BISC-1A** | **BISC-1B** | **BISC-1C** |
| **CE1176** | >20 | 0.113 | 0.183 | | | 0.113 | >20 | 0.013 | 0.019 | 0.018 |
| **Zm651** | >20 | >20 | 6.347 | | | >20 | >20 | 0.280 | 0.277 | 0.311 |
| **x2278** | >20 | 0.185 | 1.112 | | | 0.129 | >20 | 0.026 | 0.033 | 0.047 |
| **BG505** | 0.025 | 0.162 | 0.635 | | | 0.041 | 0.108 | 0.021 | 0.022 | 0.021 |
| **CH119** | 0.566 | 0.317 | 2.021 | | | 0.152 | 7.170 | 0.140 | 0.231 | 0.246 |
| **BJOX2000** | 0.014 | 0.092 | 0.354 | | | 11.405 | 0.052 | 0.022 | 0.040 | 0.027 |
| **25710** | 0.030 | 0.536 | 2.108 | | | 0.633 | 0.850 | 0.023 | 0.026 | 0.032 |
| **PV04** | 1.383 | 0.927 | 2.267 | | | 0.124 | 11.500 | 0.356 | 0.382 | 0.305 |
| **TRO11** | >20 | 0.271 | 0.102 | | | 0.185 | >20 | 0.123 | 0.331 | 0.098 |
| **CNE8** | >20 | >20 | >20 | | | 0.161 | >20 | >20 | >20 | 0.076 |
| **CNE55** | 0.023 | >20 | >20 | | | >20 | 0.589 | >20 | >20 | 5.785 |
| **x1632** | 0.007 | >20 | >20 | | | >20 | 0.056 | 0.750 | 0.168 | 0.748 |
| **246F3** | >20 | >20 | >20 | | | 0.026 | >20 | >20 | >20 | 0.017 |
| **398F1** | >20 | 0.161 | 0.157 | | | 0.074 | >20 | 0.193 | 0.165 | 0.108 |
| **CE0217** | >20 | 0.134 | 0.114 | | | >20 | >20 | 0.080 | 0.035 | 0.168 |
