# Supplementary material for: A Bispecific Antibody That Simultaneously Recognizes the V2- and V3-Glycan Epitopes of the HIV-1 Envelope Glycoprotein Is Broader and More Potent than Its Parental Antibodies
Source: mBio. 2020 Jan 14;11(1):e03080-19. doi: 10.1128/mBio.03080-19 (PMC6960291; doi:10.1128/mBio.03080-19)
Supplement: TABLE S4 [file mBio.03080-19-st004.docx]

**Table S4. IC_80_ Values (µg/mL) of BISC-1A Compared to Models**. 50:50 mix refers to equal mixtures of 10 µg/mL of each parental component. Bliss-Hill and Additive models were calculated using CombiNAber online tool.

|  | **BISC-1A** | **50:50 Mix** | **Bliss-Hill Model** | **Additive Model** |
| --- | --- | --- | --- | --- |
| **CE1176** | 0.005 | 0.028 | 0.030 | 0.077 |
| **Zm651** | 0.089 | 0.437 | 1.915 | 1.748 |
| **x2278** | 0.009 | 0.014 | 0.020 | 0.037 |
| **BG505** | 0.007 | 0.005 | 0.007 | 0.013 |
| **CH119** | 0.054 | 0.091 | 0.061 | 0.092 |
| **BJOX2000** | 0.008 | 0.013 | 0.007 | 0.008 |
| **25710** | 0.007 | 0.006 | 0.020 | 0.021 |
| **PV04** | 0.137 | 0.191 | 0.257 | 0.323 |
| **Tro11** | 0.046 | 0.097 | 0.078 | 0.078 |
| **CNE8** | >20 | 2.750 | 17.450 | 9.319 |
| **CNE55** | 2.961 | 0.012 | 0.036 | 0.036 |
| **x1632** | 0.084 | 0.005 | 0.006 | 0.006 |
| **246F3** | 17.300 | 0.724 | 12.180 | 7.570 |
| **398F1** | 0.101 | 0.067 | 0.056 | 0.056 |
| **CE0217** | 0.027 | 0.069 | 0.099 | 0.099 |
