# Supplementary material for: A Bispecific Antibody That Simultaneously Recognizes the V2- and V3-Glycan Epitopes of the HIV-1 Envelope Glycoprotein Is Broader and More Potent than Its Parental Antibodies
Source: mBio. 2020 Jan 14;11(1):e03080-19. doi: 10.1128/mBio.03080-19 (PMC6960291; doi:10.1128/mBio.03080-19)
Supplement: TABLE S5 [file mBio.03080-19-st005.docx]

**Table S5. IC_50_ Values (µg/mL) of PGT145 Bispecific Constructs.**

| **Average IC_50_** | | | | | | | | |
| --- | --- | --- | --- | --- | --- | --- | --- | --- |
|  | **PGT145** | **10-1074** | **PGT121** | **PGT128** | **PGT145 scFv-Fc** | **BISC-2A** | **BISC-2B** | **BISC-2C** |
| **CE1176** | >20 | 0.01999 | 0.020555 | 0.02862 | >20 | 0.029385 | 0.0253 | 0.02662 |
| **Zm651** | >20 | 0.07171 | 0.064095 | >20 | >20 | 0.058485 | 0.54025 | >20 |
| **x2278** | 0.006197 | 0.015245 | 0.01544 | 0.007782 | 0.023065 | 0.03661 | 0.15575 | 0.008005 |
| **BG505** | 0.026655 | 0.01347 | 0.015945 | 0.008027 | 0.075405 | 0.052945 | 0.10209 | 0.008425 |
| **CH119** | 0.4662 | 0.075205 | 0.10695 | 0.09315 | 1.7715 | 0.0552 | 0.242255 | 0.04325 |
| **BJOX2000** | 20 | 0.01745 | 0.028575 | 0.042995 | >20 | 0.02603 | 0.16373 | 0.03691 |
| **25710** | 0.007045 | 0.00599 | 0.010393 | 0.005949 | 0.05487 | 0.07763 | 0.130895 | 0.02489 |
| **PV04** | 0.3257 | 0.18745 | 0.37965 | 0.048945 | 1.0739 | 0.16955 | 4.2039 | 0.020075 |
| **TRO11** | 0.051605 | 0.0383 | 0.03521 | 0.03865 | 0.1213 | 0.033845 | 0.01631 | 0.03709 |
