# Supplementary material for: A Bispecific Antibody That Simultaneously Recognizes the V2- and V3-Glycan Epitopes of the HIV-1 Envelope Glycoprotein Is Broader and More Potent than Its Parental Antibodies
Source: mBio. 2020 Jan 14;11(1):e03080-19. doi: 10.1128/mBio.03080-19 (PMC6960291; doi:10.1128/mBio.03080-19)
Supplement: TABLE S2 [file mBio.03080-19-st002.docx]

**Table S2. IC_80_ Values (µg/mL) of Cap256.VRC26.25 Bispecific Constructs.**

|  | **IC_80_s** | | | | | | | |
| --- | --- | --- | --- | --- | --- | --- | --- | --- |
|  | **CAP256.**  **VRC26.25** | **10-1074** | **PGT121** | **PGT128** | **CAP256.**  **VRC26.25 scFv-Fc** | **BISC-1A** | **BISC-1B** | **BISC-1C** |
| **CE1176** | 7.640 | 0.077 | 0.077 | 0.042 | 11.700 | 0.005 | 0.009 | 0.007 |
| **Zm651** | 16.830 | 1.915 | 1.579 | >20 | >20 | 0.089 | 0.082 | 0.120 |
| **x2278** | 0.029 | 0.069 | 0.361 | 0.033 | 0.078 | 0.009 | 0.012 | 0.013 |
| **BG505** | 0.003 | 0.076 | 0.212 | 0.016 | 0.016 | 0.007 | 0.008 | 0.008 |
| **CH119** | 0.069 | 0.106 | 0.740 | 0.086 | 0.708 | 0.054 | 0.081 | 0.096 |
| **BJOX2000** | 0.004 | 0.032 | 0.136 | 0.086 | 0.011 | 0.008 | 0.012 | 0.011 |
| **25710** | 0.003 | 0.186 | 0.484 | 0.058 | 0.024 | 0.007 | 0.008 | 0.009 |
| **PV04** | 0.121 | 0.374 | 0.713 | 0.044 | 2.387 | 0.137 | 0.143 | 0.477 |
| **TRO11** | >20 | 0.078 | 0.043 | 0.076 | >20 | 0.046 | 0.091 | 0.027 |
| **CNE8** | 1.416 | >20 | >20 | 0.069 | 17.450 | >20 | 7.617 | 0.024 |
| **CNE55** | 0.003 | >20 | >20 | >20 | 0.036 | 2.961 | 2.172 | 0.229 |
| **x1632** | 0.002 | >20 | >20 | >20 | 0.006 | 0.084 | 0.027 | 0.060 |
| **246F3** | 1.403 | >20 | >20 | 0.013 | 12.180 | 17.300 | 8.601 | 0.006 |
| **398F1** | >20 | 0.056 | 0.069 | 0.016 | >20 | 0.101 | 0.088 | 0.031 |
| **CE0217** | >20 | 0.099 | 0.047 | >20 | >20 | 0.027 | 0.022 | 0.064 |
