# Supplementary material for: A Bispecific Antibody That Simultaneously Recognizes the V2- and V3-Glycan Epitopes of the HIV-1 Envelope Glycoprotein Is Broader and More Potent than Its Parental Antibodies
Source: mBio. 2020 Jan 14;11(1):e03080-19. doi: 10.1128/mBio.03080-19 (PMC6960291; doi:10.1128/mBio.03080-19)
Supplement: TABLE S6 [file mBio.03080-19-st006.docx]

**Table S6. IC_80_ Values (µg/mL) of PGT145 Bispecific Constructs.**

| **Average IC_80_** | | | | | | | | |
| --- | --- | --- | --- | --- | --- | --- | --- | --- |
|  | **PGT145** | **10-1074** | **PGT121** | **PGT128** | **PGT145 scFv-Fc** | **BISC-2A** | **BISC-2B** | **BISC-2C** |
| **CE1176** | >20 | 0.059425 | 0.03893 | 0.061675 | >20 | 0.07699 | 0.263395 | 0.04499 |
| **Zm651** | >20 | 0.1507 | 0.20315 | >20 | >20 | 0.1835 | 1.79235 | >20 |
| **x2278** | 0.01707 | 0.039165 | 0.04414 | 0.017635 | 0.070445 | 0.09155 | 0.39465 | 0.02226 |
| **BG505** | 0.21445 | 0.03604 | 0.03751 | 0.01999 | 0.77725 | 0.11635 | 0.26455 | 0.01733 |
| **CH119** | 5.606 | 0.2987 | 0.464 | 0.3667 | 11.27 | 0.24439 | 1.304865 | 0.09648 |
| **BJOX2000** | >20 | 0.04755 | 0.506865 | 0.10795 | >20 | 0.0591 | 0.3918 | 0.11505 |
| **25710** | 0.1417 | 0.020055 | 0.023545 | 0.015045 | 0.6532 | 0.20785 | 0.36365 | 0.0727 |
| **PV04** | 0.728 | 0.4885 | 0.7612 | 0.10725 | 2.776 | 0.4015 | 0.6072 | 0.05432 |
| **TRO11** | 0.11535 | 0.0906 | 0.076305 | 0.087455 | 0.3646 | 0.0767 | 0.04231 | 0.089305 |
