# Supplementary material for: A Bispecific Antibody That Simultaneously Recognizes the V2- and V3-Glycan Epitopes of the HIV-1 Envelope Glycoprotein Is Broader and More Potent than Its Parental Antibodies
Source: mBio. 2020 Jan 14;11(1):e03080-19. doi: 10.1128/mBio.03080-19 (PMC6960291; doi:10.1128/mBio.03080-19)
Supplement: TABLE S7 [file mBio.03080-19-st007.docx]

**Table S7. IC_95_ Values (µg/mL) of PGT145 Bispecific Constructs.**

| **Average IC_95_** | | | | | | | | |
| --- | --- | --- | --- | --- | --- | --- | --- | --- |
|  | **PGT145** | **10-1074** | **PGT121** | **PGT128** | **PGT145 scFv-Fc** | **BISC-2A** | **BISC-2B** | **BISC-2C** |
| **CE1176** | >20 | 0.16665 | 0.092425 | 0.14445 | >20 | 0.13555 | 0.76625 | 0.1195 |
| **Zm651** | >20 | 1.1086 | 0.46615 | >20 | >20 | 8.71 | 10.3005 | >20 |
| **x2278** | 0.08031 | 0.097275 | 0.10946 | 0.057225 | 0.35515 | 0.2122 | 1.1306 | 0.08512 |
| **BG505** | 6.8845 | 0.12325 | 0.12265 | 0.055815 | >20 | 0.37475 | 0.64535 | 0.03305 |
| **CH119** | 19.045 | 0.5648 | 0.6878 | 0.6589 | >20 | 0.3855 | 1.6935 | 0.26595 |
| **BJOX2000** | >20 | 0.13985 | 0.17365 | 0.4453 | >20 | 0.137 | 1.0536 | 2.6915 |
| **25710** | 6.021 | 0.0797 | 0.0861 | 0.05344 | 20 | 0.44605 | 0.99595 | 0.15335 |
| **PV04** | 2.265 | 0.9185 | 2.263 | 0.2084 | 7.899 | 0.858 | 2.4 | 0.1208 |
| **TRO11** | 0.3741 | 0.2437 | 0.15995 | 0.2165 | 0.78055 | 0.1682 | 0.1129 | 0.19715 |
